# Design and In Vitro Validation of an Orthopaedic Drill Guide for Femoral Stem Revision in Total Hip Arthroplasty

**DOI:** 10.1109/JTEHM.2024.3365300

**Published:** 2024-02-12

**Authors:** Jan-Willem Klok, Jessica Groenewegen, Olivier Temmerman, Niels Van Straten, Bart Van Straten, Jenny Dankelman, Tim Horeman

**Affiliations:** Department of BioMechanical EngineeringDelft University of Technology 2860 2628 CD Delft The Netherlands; SIMtoCARE 3633 AK Vreeland The Netherlands; Noordwest Hospital 1815 JD Alkmaar The Netherlands; Van Straten Medical B.V. 3454 PV Utrecht The Netherlands

**Keywords:** Drill positioning, guiding mechanisms, medical device prototyping, novel design, sustainable design, total hip arthroplasty

## Abstract

Objective: Cemented total hip arthroplasty (THA) demonstrates superior survival rates compared to uncemented procedures. Nevertheless, most younger patients opt for uncemented THA, as removing well-fixed bone cement in the femur during revisions is complex, particularly the distal cement plug. This removal procedure often increases the risk of femoral fracture or perforation, haemorrhage and weakening bone due to poor drill control and positioning. Aim of this study was to design a novel drill guide to improve drill positioning. Methods and procedures: A novel orthopaedic drill guide was developed, featuring a compliant centralizer activated by a drill guide actuator. Bone models were prepared to assess centralizing performance. Three conditions were tested: drilling without guidance, guided drilling with centralizer activation held, and guided drilling with centralizer activation released. Deviations from the bone centre were measured at the entry and exit point of the drill. Results: In the centralizing performance test, the drill guide significantly reduced drill hole deviations in both entry and exit points compared to the control ( $p < 0.05$). The absolute deviation on the exit side of the cement plug was 10.59mm (SD 1.56) for the ‘No drill guide‘ condition, 3.02mm (SD 2.09) for ‘Drill guide – hold‘ and 2.12mm (SD 1.71) for ‘Drill guide – release‘. The compliant drill guide centralizer significantly lowered the risk of cortical bone perforation during intramedullary canal drilling in the bone models due to better control of the cement drill position. Clinical and Translational Impact Statement: The drill guide potentially reduces perioperative risks in cemented femoral stem revision. Future research should identify optimal scenarios for its application.

## I. Introduction

Total hip arthroplasty (THA) is applied in patients with a damaged hip joint mostly due to osteoarthritis. The damaged joint is replaced with a prosthesis. The reported success rate of THA is greater than 95% in patients older than 75 years for a follow-up of 10 years [1]. However, the number of revised THA is growing due to increasing life expectancy, higher activity levels of the patients, and younger patients undergoing a primary THA. Reasons for revision peri prosthetic joint infections (PJI), loosening of one -or both components of a prosthesis and periprosthetic fractures [1], [2]. Cemented THA are favourable due to a higher survival rate compared to uncemented THA [3], [4]. However, the majority of primary THA in patients younger than 70 years, are uncemented [3]. This is mostly that a cemented prosthesis is more time consuming compared to an uncemented THA and survival rates of THA in the younger patient population is nearly similar to the cemented THA.

**FIGURE 1.**
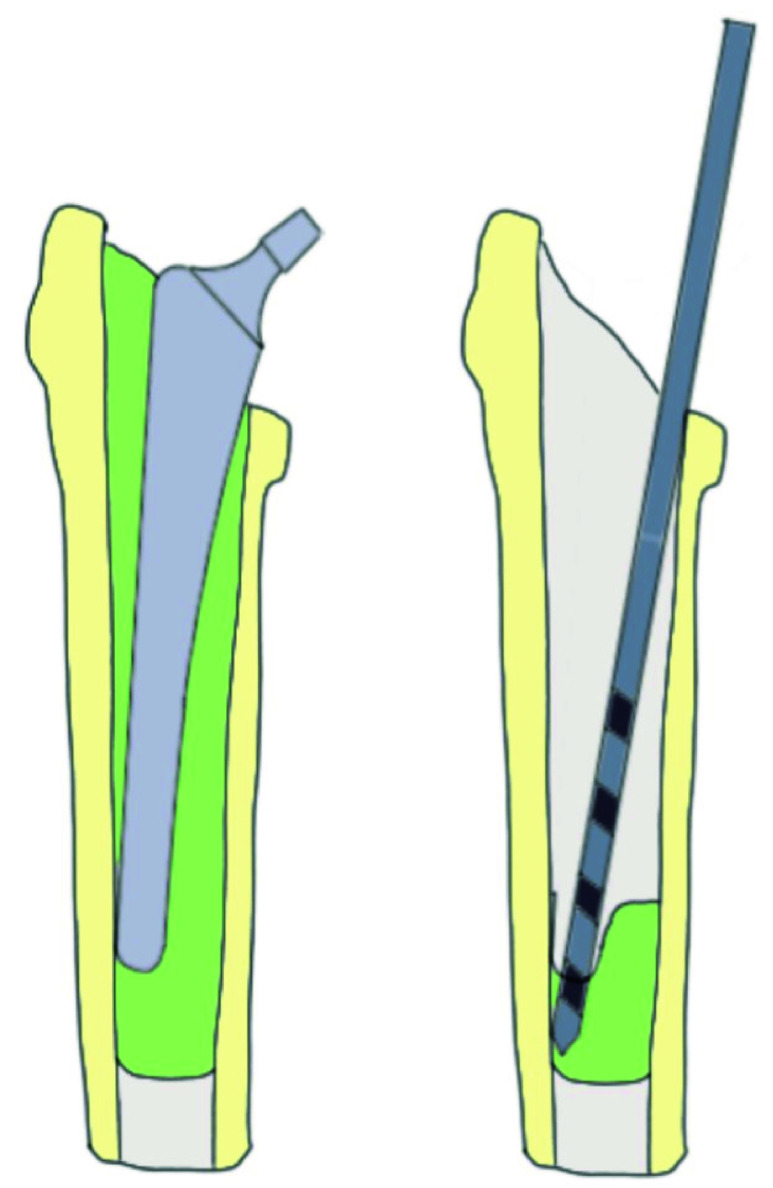
Eccentric prosthesis tip orientation in the femur. (left), increasing the risk of cortical bone damage during drilling (right).

In addition, in THA revision surgery the removal of well-fixed bone cement in the femur is a time-consuming and technically challenging procedure, especially the removal of the distal cement plug [5], [6], [7], [8]. The procedure of cement removal has a high risk of fracturing or perforating the femur, which increases blood loss and decreases bone strength [8], [9], [10], [11]. Fracture due to cortical perforation occurs most often during cement removal [12]. The risk of cortical bone perforation is increased when the intramedullary diameter is small or when the orientation of the prosthesis tip relative to the femur is eccentric. An eccentric orientation causes an oblique surface of the cement plug which makes it difficult to keep a drill in the centre of the intramedullary canal of the femur (Figure 1). The orientation and positioning of the tip depends on the anatomy of the femur and the surgical approach of the THA. Due to a lack of control of the forces that are acting on the tip during drilling, the anterolateral approach results most likely in a deviation of the tip towards the posterolateral cortex. In the posterior approach is the chance of a neutral tip position higher. If there is still a deviation of the tip in the posterior approach it is most likely towards the anterolateral cortex [8]. Figure 1 shows a deviation of the tip towards the lateral cortex. In order to reduce cortical bone perforation and subsequent complications during the cement removal phase of THA, the drill could be centred in the femoral medullary cavity.

**FIGURE 2.**
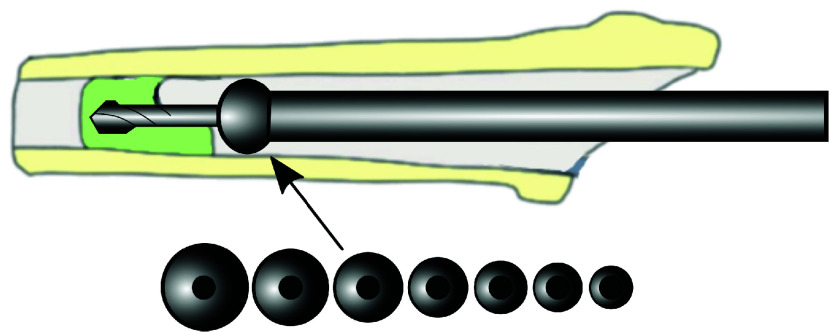
Drill guide with replaceable centre rings.

In the past, orthopaedic drill guides prototypes have been developed. In a design and validation study of Jingushi et al., [13], metal donut-shaped rings of various sizes were used to centralize the drill (Figure 2). Depending on the local femoral medullary cavity diameter, the appropriate centralizer donut size was selected preoperatively based on a femur radiograph. The centralizer was used in four THA‘s and complication rates (intra- and postoperative) were compared to 20 cases without centralizer use to validate non-inferiority. No complications were reported in the four THA‘s with a centralizer. In the THA‘s without a centralizer, the postoperative radiographs showed that a part of the cement remained in the canal of 13 patients (65%). In 5 patients (20%), perforation had occurred. However, it was not validated whether the centralizer reduced the drill deviation in the femur nor how efficient and intuitive it was to use. Also, the used centre ring was not adaptable in size, requiring an elaborate set of centre rings with varying diameter corresponding with the local femoral medullary cavity diameter, disrupting the THA‘s workflow. To improve usability and reduce impact on pre- and intraoperative THA workflow, a femoral drill guide centralizer should be adaptable to different femoral medullary cavity diameters. Therefore, a drill guide concept has been developed in collaboration with Van Straten Medical B.V. (Utrecht-De Meern, the Netherlands) and Olivier Temmerman, an orthopaedic surgeon with 14 years of experience. During this study, a final prototype of the drill guide concept is produced and its centralizing performance is evaluated and presented. For the new fixation system, a list of design requirements was established, taking into account the most important technical and clinical aspects:

1) When the centralizer is activated, the drill should reduce the risk of cortical bone perforation.
2) The drill guide is suitable for intramedullary canals with a width from 8mm to 24mm at the isthmus. The minimum 8mm diameter follows from isthmus width measurements [14]. The maximum 24mm width is chosen based on the expertise of an orthopaedic surgeon with 25 years of experience in this field.
3) The centralizer pressure on the cortical bone is less than 23MPa to avoid cortical bone damage [15].
4) The system should be modular for ease of cleaning inspection. It should take maximal 5 minutes to either assemble or disassemble the system.
5) The system, including the deforming parts should not fail during repositioning and actuation for 10 attempts.
6) All non-deforming parts should be reusable.
7) The system should cost less than €50,- to manufacture.

Particularly in the field of minimally invasive surgery, the conventional approach used to manufacture complex surgical instruments often results in instrument assemblies that are nearly impossible to inspect and require complex handling for cleaning and maintenance. As a result, most instruments are disposable and therefore, a potential financial burden for the hospitals. To gain acceptance of future technical innovation in the operating room (OR) of a more sustainable hospital, a different design approach is often required [16] leading to an intuitive and maintenance-friendly instrument that can also be used by surgeons working in poorly resourced hospitals with central sterilization departments (CSSD‘s) having limited financial budgets as compared to western hospitals. Therefore, a ‘Bare-Minimum Design (BMD)‘ methodology was used, with a strong focus on component interaction and adding functions to standard components [17], [18]. In combination with a step-wise development and an evaluation plan, key users were involved to design and produce a new handle and shaft actuation mechanism.

## II. Methodology

### A. Centralizer Concept

**FIGURE 3.**
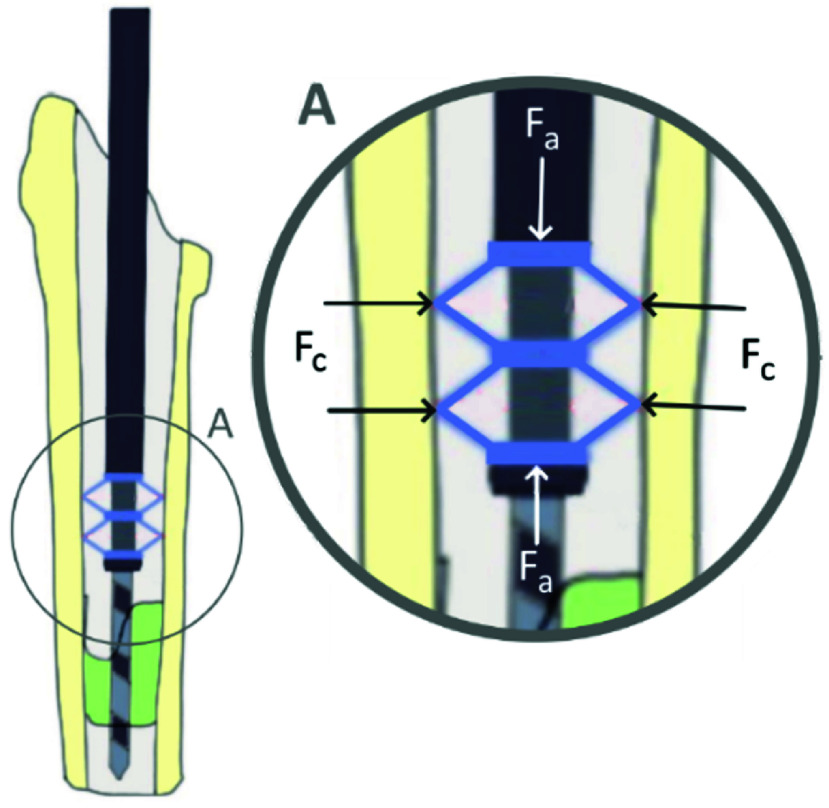
The compliant design with a centralized drill regardless of plug surface. The detail image shows a detail of the bone with an activated compliant centralizer.

**FIGURE 4.**
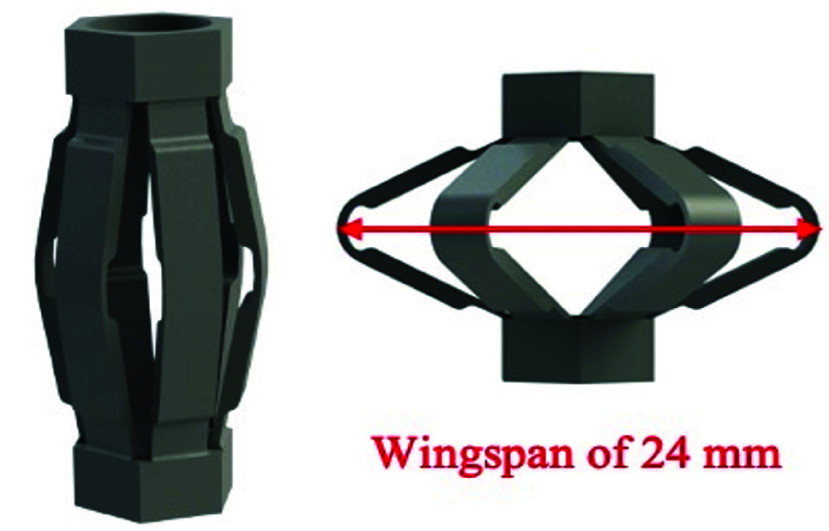
Centralizer in unactive state (left) and activated state with the maximum wingspan (right).

**FIGURE 5.**
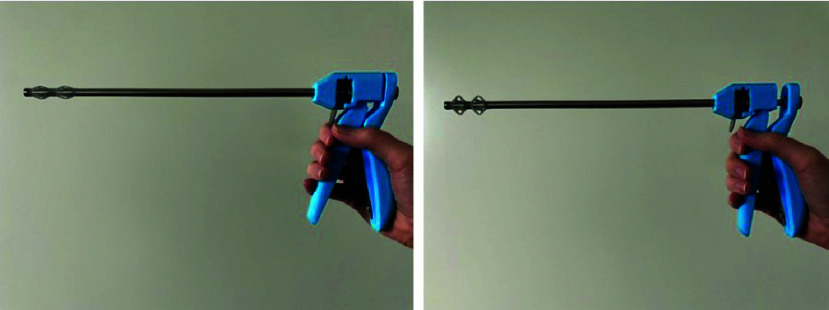
Prototype of the drill guide. Left: non-activated state; Right: activated state.

The main concept of the drill guide consists of a compliant centralizer, utilizing a radial centralizing force $F_{c}$ retain the bone drill in the centre of the medullary canal (Figure 3 and 4). The centralizer is inserted in the intramedullary canal and activated using a drill guide actuator. The bone drill passes through the actuator and the compliant centralizers. The actuator consists of a handle, an inner tube, an outer tube, and two compliant centralizers (Figure 5). In line with the BMD philosophy, the structural tube used for guiding the drill is also used for actuation of the expanding centraliser. Two centralizers are located at the distal end of the inner tube. The activation mechanism in the drill guide actuator displaces the outer tube towards the centralizers, which will expand radially when compressed in the longitudinal direction. The centralizer is a double trapezium-shaped compliant element, which elastically deforms to expand radially. When the centralizer is activated by the drill guide, the radial expansion causes the centralizer to fix itself, the drill guide actuator and the drill to the femoral medullary canal, while the drill can still rotate and move in the longitudinal direction. During cement removal, the drill guide with centralizers are placed in the medullary canal. After centralizer activation, the orthopaedic drill is inserted in the drill guide and the procedure can continue. The drill guide and centralizer cost were estimated, taking into account material, production and tooling costs, based on a mass produced series of 1000 drill guide instruments.

### B. Fatigue Test

The centralizer and the drill guide should not show structural damage during and after repositioning and actuation for 10 attempts. Therefore, the system was subjected to a fatigue test. During the test, a centralizer was placed in the drill guide and activated 10 consecutive times. The test was repeated with 5 centralizers. After testing all areas were inspected by two people.

### C. Radial Force Test

**FIGURE 6.**
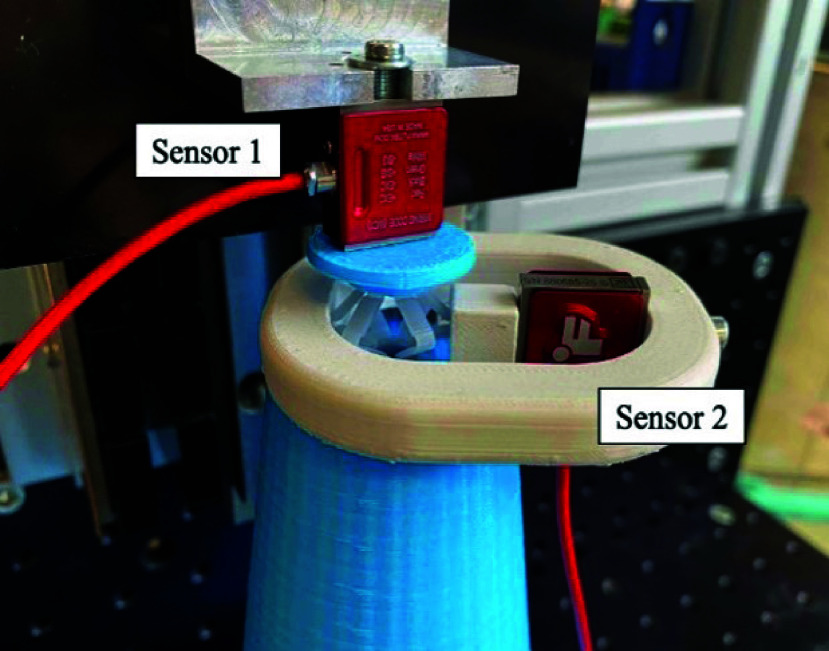
Radial force test setup showing how the sensor measures the force exerted by the expanding centralizer. Sensor 1 measures the axial force and Sensor 2 measures the output (radial centralizing) force.

Bone damage caused by the radial centralizer force exerted on the bone cortex should be prevented while using the drill guide. Therefore, the yield stress of cortical bone being 23MPa in the radial direction [15] should not be exceeded. A test setup was built to measure the radial force when the centralizer is activated (Figure 5). Two diameters ( $15mm$ and $21mm$) of intramedullary canals were simulated with two sizes of force sensor chambers. The inner diameter of the samples that were used in the centralizing performance validation is $15mm$. The diameter of the wings of the centralizers is $21mm$ when are at an angle of 45 degrees relative to the radial direction and have the highest force transmission in the radial direction. The input (actuation) and output (radial, centralizing) force was measured with two load cells (Futek, LSB201, Irvine, CA, USA) on a linear stage (ACT115 Aerotech, Pittsburgh, PA, USA). The force measurement was repeated ten times. The input force was measured by sensor 1 and was attached to the linear stage (Figure 6). Sensor 2 was attached to the chamber and measured the output force in the radial direction of two opposite wings. This is a worst case scenario, where all forces were subjected to two wings of one centralizer instead of divided over six wings and two centralizers. A clinical situation in which all radial forces are subjected to two wings of the centralizer instead of all six is in an extremely oval-shaped femoral cavity. With two wings, the contact surface area is $6mm^{2}$. Therefore, the radial force should not exceed $138N$ to prevent bone damage.

### D. Assembly and Disassembly Time

The drill guide was disassembled and assembled by five test subjects. The test subjects had no experience in similar procedures. The disassembly and assembly of the drill guide had been demonstrated once. The test subjects repeated the same procedures three times. During the test, time of disassembly and assembly of the instrument was measured.

### E. Centralizing Performance Test

**FIGURE 7.**
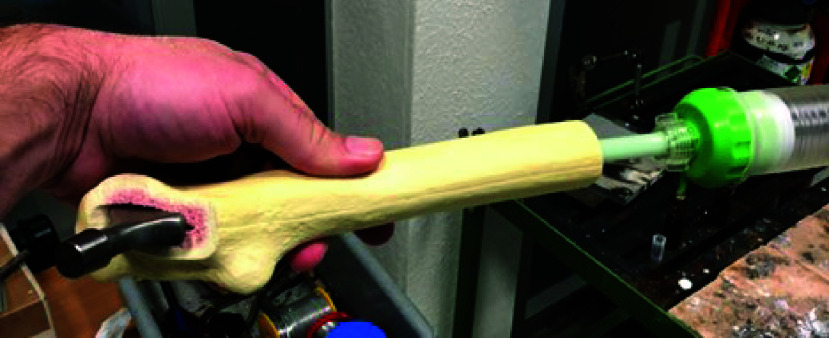
Bone model, cement and hip implant preparation. The cement is injected in the bone cavity via a syringe.

To validate whether the drill guide is reducing the deviation from the plug centre, a drill test was performed on bone models of the femur. Fifteen identical samples of orthopaedic left femur bone models with a foam cortical shell (Sawbones Europe AB, Malmö, Sweden) were prepared by an orthopaedic surgeon to mimic the physiological geometry during a THA. The femoral head and neck were removed and foam in the intramedullary canal was modified with an awl and reamers, until all femur samples had a intramedullary diameter of 15mm. The intramedullary canal was prepared for a prosthesis (EMPHASYS™, DePuy Synthes, West Chester, PA) with its tip placed against the posterolateral cortex of the femur, a surgically challenging scenario. The prosthesis was placed in the bone sample when the bone cement (Heraeus, PALACOS, Hanau, Germany) was injected as shown in Figure 7. The cement plug had an angled surface due to the deviation of the prosthesis tip.

**FIGURE 8.**
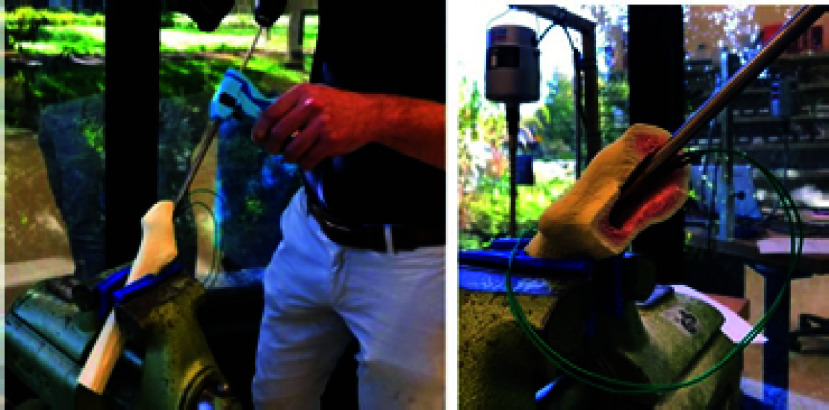
Execution of the centralizing performance validation.

From the tip of the prosthesis, the samples were cut at a length of $34mm$, which is the mean plug depth [14]. The test setup is shown in Figure 8. The orientation of the bone samples were based on a hip revision surgery.

During the test, the surgeon drilled a hole in the cement plug under three different conditions. The first condition was drilling without using the drill guide (‘No drill guide‘ condition), the current surgical standard. The second was drilling with the drill guide whereby the surgeon kept pressing the centralizer actuation handle (‘Drill guide – hold‘ condition). In the third condition, drilling was also performed with the drill guide but the surgeon released the actuation handle during drilling after the centralizers were activated prior to the drilling (‘Drill guide – release‘ condition). The two latter conditions are plausible drill guide user cases. Each condition was repeated five times, and the total of 15 drilling trials were performed in a randomized order. The inner tube and the drill bit were cleared from cement grit and new centralizers were used for each trial.

After drilling, the samples were cut at a length of $55mm$ from the bottom to visualize the drill hole entry and exit for analysis. Both sides were photographed by holding the samples perpendicular against a transparent plate with a tape measure. The photographs were analysed in ImageJ (NIH, Bethesda, MD, USA) in which the x- and y-coordinates of the centroids of the cement plug and drilled hole relative to the femoral medullary cavity centre were determined. From this, the absolute deviation was calculated. A Mann-Whitney U test was performed to test the statistical significance, because the deviation data was not normally distributed.

## III. Results

### A. Design

A functional prototype of the drill guide was built (Figure 5). All of the non-deforming parts are reusable. When the centralizers are activated, their wingspan position is retained. The centralizers are deactivated by a release trigger. The centralizer design is a compliant mechanism with three living hinges in every wing. The prototypes of the centralizers were made of a Tough 1500 Resin because this resin simulates the elasticity and strength of polypropylene (PP). The prototypes were 3D printed using a stereolithography (SLA) printer (Formlabs, Form 3, Boston, MA, USA). The drill guide and centralizer cost was estimated at €46.60 (see Supplemental A for a cost breakdown).

### B. Fatigue Test

None of the centralizers broke or were damaged during or after the fatigue test according to the two observers. The drill guide did not fail during repositioning and reactivation.

### C. Radial Force Test

The maximum radial load at a wingspan of $15mm$ and $21mm$ was $13.6N$ (SD $1.4N$) and $12.1N$ (SD $0.8N$) respectively and did not exceed 138N.

### D. Assembly and Disassembly Time

**FIGURE 9.**
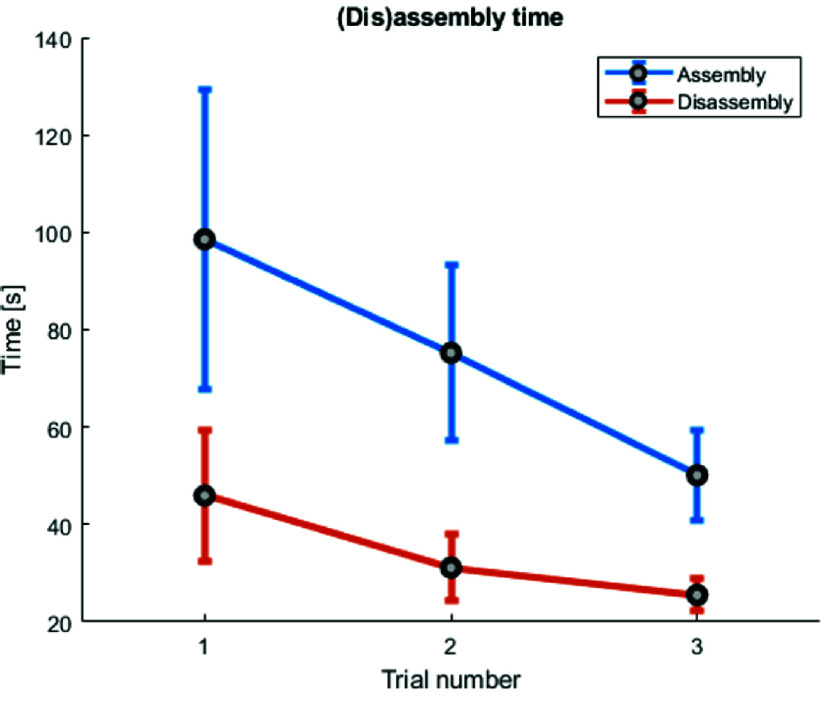
Mean drill guide assembly and disassembly time with standard deviation (SD).

The total disassembly and assembly time results are shown in Figure 9. The average disassembly time over three trials was $34.2s$ (SD $12.2s$). The average assembly time was $74.6s$ ( $SD 28.4s$). Both times show a learning curve.

### E. Centralizing Performance Test

**FIGURE 10.**
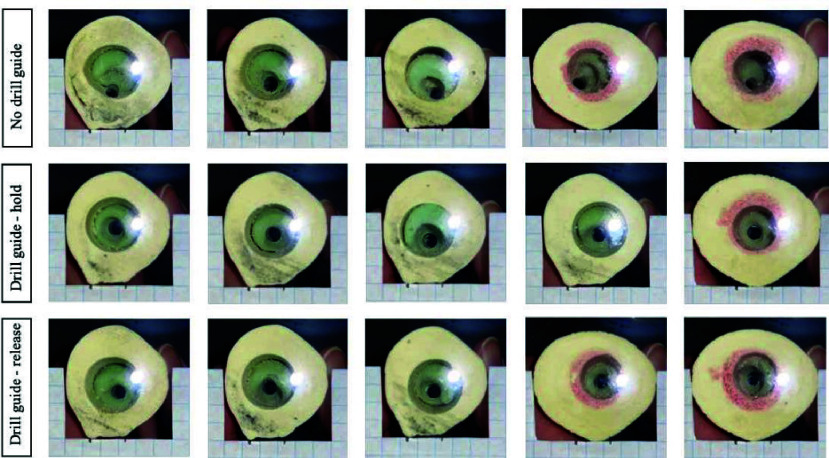
Drill hole locations at the cement plug entry point separated for the tree different conditions.

**FIGURE 11.**
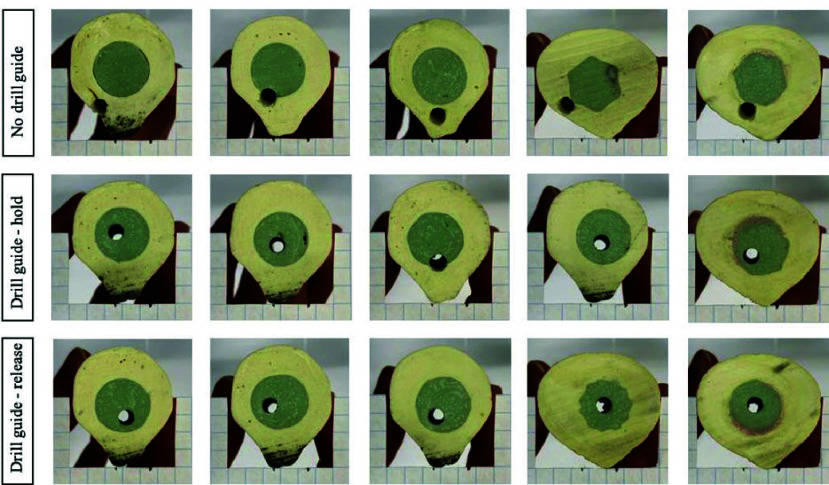
Drill hole locations at the cement plug exit point separated for the tree different conditions.

**FIGURE 12.**
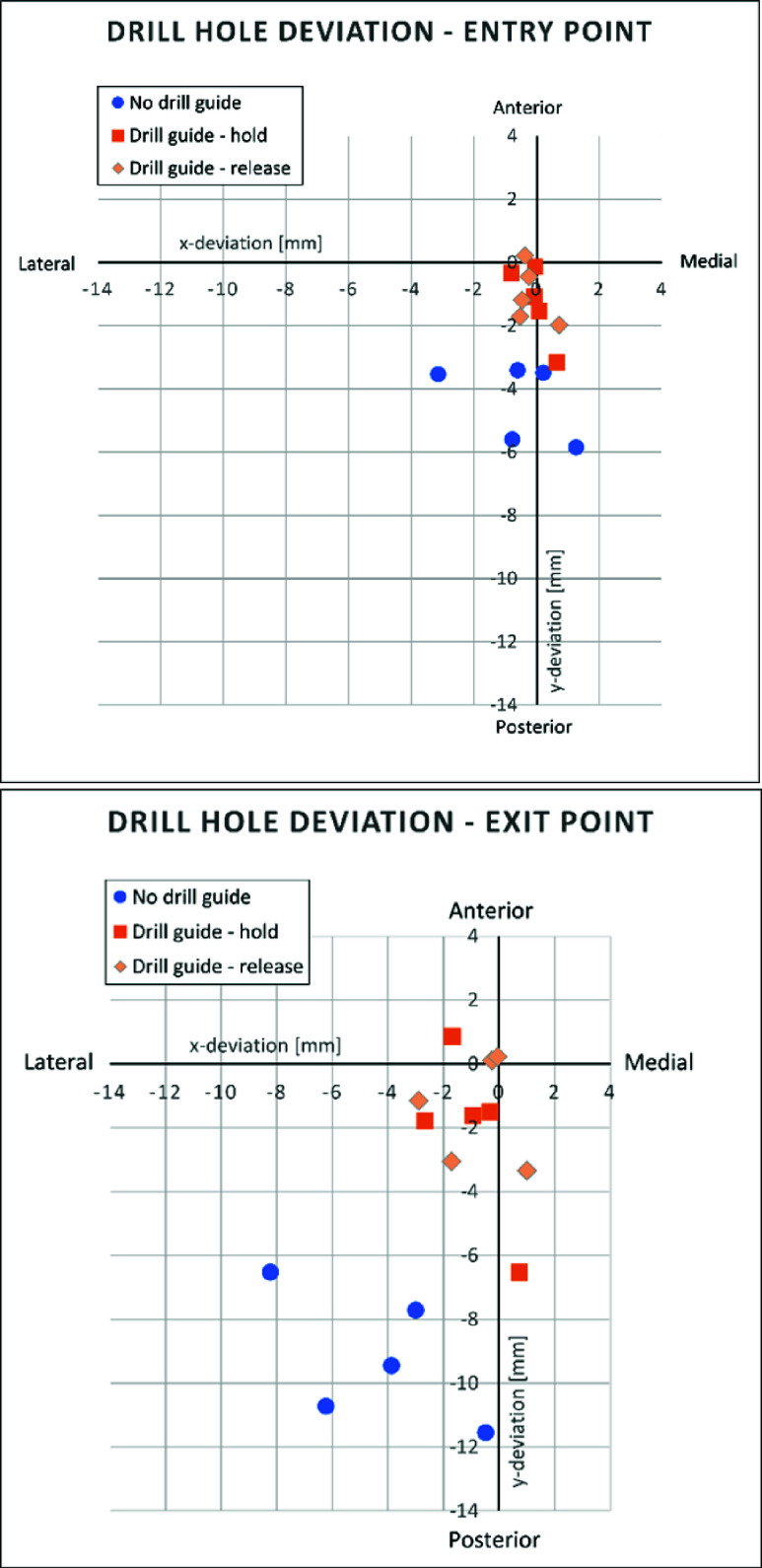
Scatter plots of the drill entry (top) and exit (bottom) deviation relative to the femoral medullary cavity.

Photograph results of the drill entry and exit points of the drill holes in the bone phantom are presented in Figure 10 and 11. Figure 12 shows the respective deviation scatter plots of the drilled hole relative to the plug centre at the entry and exit site. In Table 1, the results of the $x$ and $y$ deviation are reported (means and standard deviations). During one ‘Drill guide – hold‘ test, the centralizers failed. This test resulted in an absolute deviation of $6.6mm$ at the exit point and $3.2mm$ at the entry point. Results of the Mann-Whitney U test are shown in Table 2. The absolute deviation of the validation of the drill entry and exit points with conditions ‘Drill guide – hold‘ and ‘Drill guide – release‘ were significantly lower than the ‘No drill guide‘ condition (both $p < 0.05$). There was no significant difference between the tests where the drill guide was kept compressed and the drill guide was released, this holds for the entry side ( $p=0.9$) as well as the exit side ( $p=0.8$). After the fifteen drilling trials, the drill guide showed no signs of wear-induced loss of functionality.

**TABLE 1:** Mean and Standard Deviation of the Drill Entry and Exit Point Relative to the Femoral Medullary Cavity

Group	Entry point deviation		Exit point deviation	
	Mean (mm)	SD (mm)	Mean (mm)	SD (mm)
No drill guide (control)	4.7	1.2	10.6	1.6
Drill guide – hold	1.4	1.2	3.0	2.1
Drill guide – release	1.3	0.8	2.1	1.7

**TABLE 2:** Group Comparison for Entry and Exit Points With p-Values (Mann-Whitney U Test)

Group comparison	Entry point	Exit point
	p-value	p-value
Drill guide – hold vs. No drill guide	< 0.05	< 0.05
Drill guide – release vs. No drill guide	< 0.05	< 0.05
Drill guide – hold vs. Drill guide – release	0.92	0.75

The drill guide was reused for 30 procedures without any observable wear or malfunctioning. The surgeon that conducted the centralizing performance test concluded that the centralizing procedure with the drill guide was intuitive, and appreciated the fact that after activation, the centralizer was locked by the drill guide mechanism and no continuous pressure on the handle was needed. The surgeon deemed the drill guide and centralizer system favourable for any THA‘s but especially for THA‘s with small ‘isthmus diameter or uneven cement plug surfaces.

## IV. Discussion

### A. Design

A new drill guide concept was developed based on the Bare Minimum Design method with a minimum number of components within a modular design that allows for cleaning, inspection and maintenance. The developed drill guide concept showed that a compliant, radially expanding centralizer is a viable method to significantly decrease the risk of cortical bone damage while drilling in the intramedullary canal in THA.

In the centralizing performance experiment, the drill holes showed significantly less deviation from the bone phantom centre in the Drill guide condition compared to the No drill guide condition, both in the entry and exit measurements. Therefore, Design requirement 1, stating that the drill guide should reduce risk of bone perforation, is met. In all but one of the results of the Drill guide condition, the drill did not perforate the cortical bone phantom. Therefore, the Design requirement 2, stating that the drill should not perforate the cortical bone at all is not met completely.

However, the post-experiment examination showed that in the experiment run where the bone phantom was perforated, a broken centralizer was used. As every centralizer was inspected prior to the experiment on defects, the damage must have occurred during centralizer activation in the bone phantom. Therefore, it can be assumed that the broken centralizer caused reduced centralizing performance. With the compliant hinges used in the centralizer design, there is a risk of failure after bending them several times due to material fatigue. Therefore, the decision was made to make the centralizers disposable.

The bone phantom had an intramedullary diameter of $15mm$. However, Design requirement 2 stated that the drill should not perforate the femur if the cement plug is located in an intramedullary canal with a minimum isthmus width of $9mm$. Using the drill bit diameter, the extent to which this requirement is met can be validated. A $4.7mm$ drill bit diameter translates to a maximum allowed absolute deviation of the drill of $2.15mm$ from the bone phantom centre in the radial direction for an isthmus width of $9mm$. In the Drill guide group, the maximum deviation is $3.50mm$, excluding the result of the broken centralizer. The minimum isthmus width where no cortical bone perforation occurred with an intact centralizer is $11.7mm$. Even though Design requirement 2 is not met with smaller isthmus widths, cortical bone perforation risk is still lower compared to the No drill guide group as there is a significant difference between the groups.

To prevent cortical bone damage due to the centralizer forces, bone pressure at the centralizer wings should not exceed $23MPa$. Taking into account the contact surface area of the centralizer, the radial force should not exceed $138N$. The results of the radial force test meet Design requirement 3, because the maximum radial force was $13.6N$. As a result, the centralizers will fail before they can damage the bone. The average assembly and disassembly time was $74.6s$ and $34.2s$ respectively, fulfilling the (dis)assembly time limits of Design requirement 4. The system did not fail during repositioning and actuation for a total of 10 times and the non-deforming parts were reusable, fulfilling Design requirement 5 and 6 respectively. The total per unit cost of the system is €46.60, which means that Design requirement 7 is also met.

### B. Limitations and Further Research

The disposable centralizers were 3D printed of a photopolymer resin with a stiffness similar to PP. However, the 3D printed material has a lower elongation before break compared to PP. For future prospects, it is desirable to manufacture large amounts of disposable centralizers. For this, a feasible manufacturing technique is PP injection moulding. With injection moulded PP, the fatigue test, radial force test and centralizing performance test results might show less flexure breaking. Therefore, future studies should use injection moulded PP instead of 3D printed centralizers. Realistic phantom bone models were used during the centralizing performance test. Although the bone models were anatomically correct, mimicked the cancellous cortical bone structure and are used as an alternative to human cadaver bone according to the manufacturer, deviations from bone mechanical properties are possible.

The centralizing performance experiment simulated a challenging clinical situation: the prosthesis tip was placed against the posterolateral cortex of the femur causing a cement surface that is slightly angled. This is a common occurrence [19]. Therefore, application of the drill guide should be decided based on preoperative imaging. A small isthmus width or an angled cement plug surface are indicators for drill guide use. When these factors are not present, the drill guide might offer less advantage. Still, the drill guide can reduce iatrogenic complication risk in THA in general. Future studies should focus on identifying risk factors for which the drill guide provides the most risk reduction.

### C. Impact

The absolute deviation of the drill was significantly higher during the test without the drill guide compared to the tests with the drill guide. This shows that the drill guide would have a valuable effect on centric drilling in the cement plug. Therefore, if the bone drill guidance and centraliser is successfully developed and implemented, cement removal while using the drill guide reduces the risk of cortical bone perforation. The handle parts and the centralizers are made such that they can be made from reprocessed polypropylene, reducing the carbon footprint of the drill guide [20].

## V. Conclusion

The drill guide concept significantly reduced the drill hole deviation from the bone centre in a bone model. Therefore, the concept showed that a compliant, radially expanding centralizer could be a viable method to significantly decrease the risk of cortical bone damage while drilling in the intramedullary canal in THA. Further research is needed to identify risk factors for which the drill guide provides the most risk.

## Supplementary Materials

Supplementary materials
